# Implementation of transanal minimally invasive surgery (TAMIS) for rectal neoplasms: results from a single centre

**DOI:** 10.1007/s10151-021-02556-y

**Published:** 2021-12-14

**Authors:** W. Lossius, T. Stornes, T. E. Bernstein, A. Wibe

**Affiliations:** 1 Department of Surgery, St. Olav’s Hospital, Trondheim University Hospital, Trondheim, Norway; 2 Department of Cancer Research and Molecular Medicine, Norwegian University of Science and Technology, Trondheim, Norway

**Keywords:** Rectal neoplasms, Local excision, TAMIS, Recurrence, Completion total mesorectal excision (cTME)

## Abstract

**Background:**

Local excisions are important in a tailored approach to treatment of rectal neoplasms. In cases of low risk T1 local excision facilitates rectal-preserving treatment. Transanal minimally invasive surgery (TAMIS) is the most recent alternative developed for local excision. In this study we evaluate the results after implementing TAMIS as the routine procedure for local excision of rectal neoplasms.

**Methods:**

All patients who underwent TAMIS from January 2016 to January 2020 at St. Olav’s University Hospital were included, and clinical, pathological and oncological data were prospectively registered. The primary endpoint was local recurrence, and the secondary endpoint was complications.

**Results:**

There were 76 patients (42 men, mean age was 69 years [range 26–88 years]), The mean tumour level was 82 mm (range 20–140 mm) from the anal verge measured on rigid proctoscopy, and mean tumour size was 32 mm (range 8–73 mm). Three patients experienced complications needing intervention (Clavien–Dindo > 3A). Seventeen patients had rectal adenocarcinoma, 9 of whom underwent R0 completion total mesorectal excision (cTME). Fifty-five patients had an adenoma, 3 of whom developed recurrence (5.4%) within 12 months. All recurrences were treated successfully with a new TAMIS procedure. In addition, TAMIS was used in treatment of 2 patients with a neuroendocrine tumour, 1 patient with a haemangioma and 1 patient with a solitary rectal ulcer.

**Conclusions:**

TAMIS surgery is associated with a low risk of complications and a low recurrence rate in rectal neoplasms. In cases of adenocarcinoma, R0 cTME surgery is feasible in the sub-group with high risk T1 and T2 tumours.

## Introduction

Local excision is an important part of the treatment of rectal neoplasms, most commonly used in treatment of adenomas as a supplement to endoscopic mucosal or submucosal excisions (EMR/ESD). In cases of adenoma, endoscopic excisions and local excisions by the transanal endoscopic microsurgery (TEM) technique have been shown to have comparable results [1]. For rectal adenocarcinomas, total mesorectal excision (TME) is the standard treatment [2], but in T1 tumours with low risk of lymph node metastases, local excision with TEM/TAMIS has oncological results that are comparable to TME [3–5]. Furthermore, the obvious benefits of local excision are organ-sparing, lower morbidity and mortality, and better functional results [6–8].

However, due to tumour heterogeneity and a lack of accuracy among the available diagnostic tools, discrimination between a benign and an early stage malignant tumour is a major clinical challenge [9, 10]. Thus, some patients may be overtreated by a TME procedure, and others may be undertreated by a transanal procedure.

TEM has been the gold standard in local excisions and has been proven superior to conventional transanal excisions [11]. However, following some technical limitations related to TEM, TAMIS has been introduced as an alternative since 2010. The potential benefits include more flexibility with the use of standard laparoscopic instruments and the standard lithotomy position [12]. Some authors suggest that the elastic GelPort is safer than the rigid TEM platform with respect to the risk of sphincter traction and incontinence [13]. Since the introduction of the TAMIS technique, multiple reports have documented that its safety and results are comparable to those of TEM [14, 15], although there is concern about an unacceptably high risk of involved excision margins [16].

In 2015, we replaced TEM with TAMIS, and since 2016, the results of all TAMIS procedures have been prospectively registered. Over the last 2 years we have implemented TAMIS with submucosal dissection in selected cases of large, sessile adenomas and anteriorly located tumours, where perforation of the abdominal cavity is of concern. In the era of implementation of national screening programs, the number of patients with early stage rectal cancer (ERC) and advanced adenomas is expected to increase. An evaluation of the treatment of such lesions is, therefore, increasingly important. The purpose of this study was to assess the safety of the TAMIS procedure and to evaluate the results after implementation of a new procedure.

## Materials and methods

From January 2016 to January 2020, all consecutive patients treated with TAMIS were prospectively included in the study. Rigid proctoscopy with biopsy of the lesions was performed, and tumour level was given as distance from the lower border of the lesion to the anal verge. Rectal ultrasound was not used routinely, but in case of adenocarcinoma on preoperative biopsies or clinical suspicion of malignancy, magnetic resonance imaging (MRI) was performed. The surgeries were conducted by a team of three surgeons (WL, TEB, TS), all of them specialists in colorectal surgery. One surgeon had previous experience with TEM and had conducted nine TAMIS-procedures at the start of inclusion, while the two remaining surgeons had no prior training in this procedure. Data concerning demographics, preoperative examinations, operative data, complications, histopathology and completion surgery were registered prospectively, and evaluation of recurrence was conducted retrospectively. Complications were graded by the Clavien–Dindo classification system [17].

### Surgical technique

**Fig. 1:**
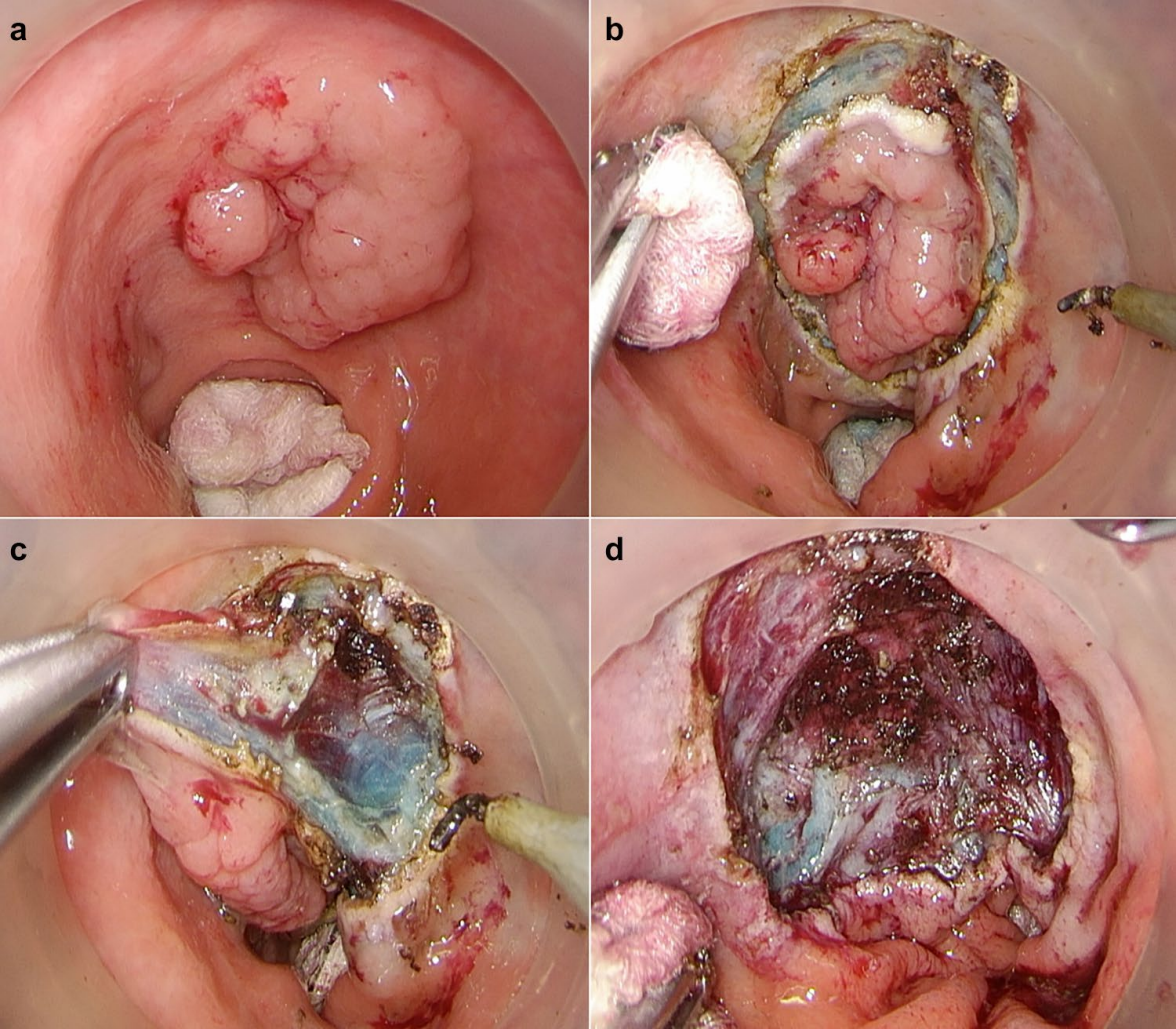
Transanal minimally invasive surgery with submucosal excision. **a** Tumour at anterior wall seven to ten cm from anal verge. **b** Mucosal incision after submucosal elevation. **c** Dissection on the muscularis propria. **d** Complete dissection

The procedure was performed under general anaesthesia using an AirSeal insufflator and a Gel Port using three ports and standard laparoscopic instruments after mechanical bowel preparation. All patients received preoperative antibiotics (doxocycline 400 mg iv + metronidazole 1500 mg iv). For lesions 3 cm or smaller, located posteriorly with underlying mesorectal fat, full-thickness excisions were preferred, while submucosal dissection was chosen with larger or anteriorly located lesions. Full-thickness excisions were closed by the use of v-lock 3–0 resorbable sutures. In submucosal dissection procedures, the submucosal plane was elevated by injection of adrenaline/mannitol/blue dye and dissection was conducted by monopolar hook diathermy on the muscularis propria layer. These defects were not closed (Fig. 1). In cases of full-thickness dissection, patients were given antibiotics orally or intravenously for 3 days after their surgeries depending on the size of the excision/defect.

### Follow-up

All benign polyps were controlled by proctoscopy after 3 and 12 months. After 12 months the patients were referred to further colonoscopy controls according to national guidelines.

All patients with a rectal cancer treated by TAMIS who did not undergo completion TME surgery were followed up with a proctoscopy and MRI every 3 months, and a computed tomography (CT) scan every 6 months the during first year, and thereafter every 6 months in accordance with national guidelines [18].

The primary outcome of the study was local recurrence, and secondary outcomes were feasibility of completion TME surgery, rate of positive excision margins, and complications.

The study was approved by the regional ethics committee.

## Results

Seventy-six patients, (42 men [55%] and 34 women [45%], mean age was 69 years [(range 26–88 years]), underwent a TAMIS procedure during the study period. Mean tumour level was 82 mm (range 20–140 mm, SD 27), and mean tumour size was 32 mm (range 8–73 mm, SD 14). All operations were successfully completed by TAMIS without needing conversion to abdominal surgery.

The indication for TAMIS was adenoma (*n* = 55), adenocarcinoma (*n* = 17), neuroendocrine tumour (*n* = 2), bleeding haemangioma (*n* = 1), and solitary rectal ulcer (*n* = 1) (Table 1). Of the 17 adenocarcinomas, 8 preoperative biopsies correctly showed adenocarcinomas, while 9 showed adenomas of various degree of dysplasia. Five patients did not have a preoperative biopsy, the patient with haemangioma and 4 with adenomas. In the group of 72 patients with either adenoma or adenocarcinoma, MRI was performed in 42 patients (58%). In 25 of 42 patients (60%) the MRI-staging was accurate in differentiating adenoma from adenocarcinoma. In the remaining 17 patients, MRI overstaged 13 patients with adenomas (31%) as adenocarcinomas, and understaged 4 patients with adenocarinomas (9%) as adenomas.

**Table 1:** Definitive pathology in 76 patients who had TAMIS excision

Pathology	*N* (%)
Adenoma	55 (72.3)
Low grade dysplasia	20
Moderate dysplasia	9
High grade dysplasia	26
Adenocarcinoma	17 (22.4%)
T1sm1	3
T1sm2	3
T1sm3	5
T2	5
T3	1
Others	4 (5.2)
Neuroendocrine tumour	2
Haemangioma	1
Solitary rectal ulcer	1

Mean operating time was 70 min (range 28–142 min). Ten patients had complications (13%). Three patients underwent a re-operation/examination under general anaesthesia (Clavien–Dindo 3B). One patient underwent a secondary suture of a suture line defect, 1 patient was examined due to bleeding, and 1 due to high C-reactive protein postoperatively, both without conclusive pathology.

The remaining 7 patients had minor complications (Clavien–Dindo grade ≤ 2), including 3 cardiac complications (congestive heart failure and arrhythmia), and 4 patients had infectious complications (urinary tract, pneumonia, and surgical site infection).

### Adenocarcinomas

All 17 patients who underwent TAMIS for an adenocarcinoma had an R0 excision. Of these, 16 patients were treated with curative intent, and 1 patient with a T3 cancer was offered a compromise local excision due to major comorbidity. Eight of these patients had confirmed malignancy in the preoperative biopsy. Results from histopathological examinations are given in Table 2.

**Table 2:** Results from histopathological examination, treatment and recurrence in 17 patients with adenocarcinoma treated with transanal minimally invasive surgery (TAMIS)

T-stadium	TAMIS final treatment	Completion surgery	Local or distant recurrence
T1sm1	3	0	No
T1Sm2	2	1	No
T1Sm3	1	4	No
T2	1	4	No
T3*	1		Yes
Total	8	9	

*Compromise strategy due to extensive comorbidity

Among the 16 patients with adenocarcinoma treated with curative intent, 9 had completion TME surgery (cTME) after approximately 6 weeks. Indications for cTME were T2 in four patients, T1sm3 in four patients, and T1sm2 in 1 patient who had extensive tumour budding on histopathology. All patients received radical, R0 TME surgery, as 5 had an abdominoperineal resection and 4 had a low anterior resection. There have been no local or distant recurrences in this group within a mean follow-up of 16 months (range 9–20 months). None of the patients had tumour remnants of the primary lesion on histopathology, but 2 patients had N + disease (both pT2). In addition, the patient with tumour budding had remnant tumour buds in the resection specimen, giving a total of 3/9 patients with tumour remnants on cTME surgery.

Eight patients underwent TAMIS for adenocarcinoma as their final treatment, (see Table 2). One patient with a T3 tumour underwent TAMIS as compromise treatment due to extensive comorbidity. Three patients with a T1sm1 tumour, 2 patients with T1sm2, 1 patient with a T1sm3 and 1 patient with a T2 tumour underwent TAMIS as their final treatment. The 4 patients with T1sm2, T1sm3 and T2 were all recommended completion TME surgery in accordance with the national guidelines, but they preferred an organ-sparing control approach instead. In this group of 8 patients with adenocarcinoma who received TAMIS as their final treatment, we observed 1 local recurrence in the patient who had a compromise TAMIS for a T3 tumour, which was treated with palliative radiotherapy by 5 × 5 Gy. There have been no local or distant recurrences within a median observation time of 28 months (range 15–43 months) in the remaining 7 patients.

### Adenomas

A total of 55 patients with advanced adenomas were treated with TAMIS, 3 of whom developed local recurrences (5.4%) during the observation time of 24 months (range 2–49 months) All recurrences appeared within 12 months and were treated with a new TAMIS procedure. Two of the recurrences occurred in cases of large tumours (55 mm and 65 mm), one of which had an R1 excision. The third recurrence occurred in a case of a 15 mm tumour with an R0 excision.

The excision margin was short (defined as ≤ 1 mm) or involved in 6 patients (10.9%). In 8 patients (14.5%) the excision margin was uncertain on histopathology due to piecemeal excisions, but macroscopically free. In the remaining 41 patients (74.5%) the margins were defined as free (margin > 1 mm) on histopathology.

## Discussion

This study supports the applicability and safety of the TAMIS procedure in treatment of rectal neoplasms, as we observed a low rate of major complications and a low recurrence rate. Considering adenomas, recurrences seem to develop early (within 12 months), and a new organ-sparing procedure is likely feasible. The present results are in line with prior findings indicating that in case of high risk features on histopathology after TAMIS for early rectal cancer, cTME surgery is feasible, achieving R0 resections and oncological results comparable to primary TME surgery [19].

### Adenoma and TAMIS

Large adenomas (size > 40 mm) are a particular challenge. In the first part of the study period we only did full-thickness excisions, and with large polyps the defects became correspondingly large. Two patients underwent reoperation, and in one of these a small defect was closed, resulting in a prolonged hospital stay. Sometimes this also resulted in piecemeal excisions as we either endoscopically or by TAMIS excised the top of the polyps to better visualize the base of large semicircular polyps, resulting in uncertain excision margins on histopathology. In the latter part of the study period we performed TAMIS with submucosal dissection in these cases, experiencing less of a narrowing tendency and even reduced complications, and we advocate this technique if possible for these types of adenomas. The high rate of piecemeal excisions (14%) in the adenoma group is also a result of undertaking large polyps up to 7 cm in size, where fragmentation of the specimen was deemed necessary to allow visualization. TAMIS was not undertaken for tumours of this size with verified or suspected adenocarcinoma.

Due to the use of diathermy, the excision margins are often short or involved on histopathological examination, 10.9% in our study, despite good visual control when dissecting. The low recurrence rate in this group of advanced adenomas may indicate that the short excision margins on histopathology do not have a strong association with recurrence due to thermal necrosis along the excision line and shortening of the neighbouring tissue.

### Adenocarcinoma and TAMIS

Patients who undergo TAMIS surgery are often a select group of patients with larger neoplasms and with a high risk of invasive cancer. In our study, 17/76 patients (22%) had cancer, which is similar to other series [20], mandating a very close follow-up regimen and a strategy for completion surgery. According to our national guidelines, patients with T1sm2 or higher, lymphovascular infiltration, low differentiation or tumour budding will require cTME. Still, in our cohort, including 17 patients with cancer, and after obtaining information about the risk of local recurrence and lymph node positive disease, 8 patients did not need or want completion, but preferred their organ-sparing procedure for cancer. It is important to remember that this tendency towards rectal-preserving treatment may impose a potential oncologic risk [21].

Although only 9 patients had cTME and the observation time is short, the study supports the findings in other studies that cTME is feasible after local excisions of early rectal cancer, and the long-term oncological results seem good as we had no recurrences, local or distant, in this group [5, 22].

In Norway postoperative radiochemotherapy (RCT) after local excisions has not been widely used, but this may be an alternative to cTME in selected cases, in particular in patients who refuse TME surgery or with severe comorbidities. The role of local excision after neoadjuvant RCT is not clearly defined and we are awaiting the results from ongoing studies on this strategy [23–25].

We observed severe complications in 3 patients (Clavien–Dindo > 3B), which in some cases necessitated reoperation. They all had large adenomas (size > 50 mm) with corresponding large defects, which can be more difficult to close. During the last 2 years we have approached these large polyps with submucosal dissection if they are found to be benign in a preoperative biopsy. This is usually feasible, and even if we get small full-thickness defects (< 0.5 cm) they are usually easy to close with sutures. We advocate this strategy in larger adenomas (> 50 mm). In cases of high grade dysplasia or adenocarcinoma, the patients with the larger neoplasms are often recommended primary TME surgery as the national guidelines in Norway do not recommend local excisions in cases of malignancy in tumours > 30 mm.

Of a total of 17 patients with adenocarcinoma, the preoperative biopsy showed adenocarcinoma in only in 8 patients, which reflects the difficulties in preoperative diagnostics in this group of patients. This is likely the result of the biopsy only showing a small sample of the tumour, where malignant features are not displayed. Due to this high risk of adenocarcinoma, we advocate the need for preoperative MRI. Although preoperative MRI has a fairly low positive predictive value [26, 27], and may not guide the clinical decisions with certainty, an MRI after local excision and before completion TME is more difficult to interpret. This study reported a low accuracy of MRI in differentiating adenomas from adenocarcinomas (60%), with a tendency to overstage rectal neoplasms (31%) while also understaging to a certain degree (9%). We do, however, find it useful in excluding early rectal cancer (T1–T2) from more advanced disease (T3 + and N1 +). Moreover, the use of ERUS may facilitate the discrimination of benign versus malignant lesions in experienced hands, but it has a low ability to identify N + disease and no ability to discriminate between Sm-levels [28].

### Limitations

This study has several limitations, the most significant of which are the short observation time, and the limited number of patients, which is due to a fairly recent implementation of TAMIS surgery. The study lacks a standardized evaluation of functional results, which might be interesting to compare with endoscopic procedures on benign cases.

In this study the use of preoperative MRI has been inconsistent in case of adenoma in preoperative biopsy, and the observed accuracy of MRI to discriminate between adenomas and early stage rectal cancer was low.

## Conclusions

TAMIS is an effective method for excision of rectal tumours with a low risk of complications and a low recurrence rate. In cases of malignancy, R0 completion TME surgery is feasible in the group with high risk T1 tumours, and for a significant proportion of patients with early rectal cancer a TAMIS procedure is a sufficient operation, facilitating organ-sparing and reduced morbidity compared to primary TME surgery.

## Data Availability

Data are stored locally and available for review on request as per national regulations.
